# What We Should Expect from an Innovative Intra-Articular Hyaluronic Acid Product: Expert Opinion Based on a Comprehensive Review of the Literature

**DOI:** 10.3390/jcm12237422

**Published:** 2023-11-30

**Authors:** Alberto Migliore, Emmanuel Maheu, Luca Saccone, Gustavo C. de Campos, Lyudmila Alekseeva, Xavier Chevalier, Thierry Conrozier, Sergio Crimaldi, Demirhan Diracoglu, Gabriel Herrero-Beaumont, Giovanni Iolascon, Ruxandra Ionescu, Jörg Jerosch, Jorge Laíns, Souzi Makri, Natalia Martusevich, Marco Matucci Cerinic, Karel Pavelka, Robert J. Petrella, Umberto Tarantino, Raveendhara R. Bannuru

**Affiliations:** 1 Rheumatology Unit, San Pietro Fatebenefratelli Hospital, 00189 Rome, Italy; 2 Rheumatology Department, AP-HP, Saint-Antoine Hospital, 75012 Paris, France; 3 Department of Orthopaedics and Traumatology, San Pietro Fatebenefratelli Hospital, 00189 Rome, Italy; 4 Department of Orthopaedics and Traumatology, Fondazione Policlinico Universitario Campus Bio-Medico of Rome, 00128 Rome, Italy; 5 Department of Orthopaedics and Traumatology, University of Campinas, Saint Paul 13083, Brazil; 6 Department of Metabolic Diseases of Bone and Joints, VA Nasonova Research Institute of Rheumatology, 115522 Moscow, Russia; 7 Department of Rheumatology, Hôpital Henri-Mondor, Université UPEC Paris XII, 94000 Paris, France; 8 Service de Rhumatologie, Hôpital Nord Franche, 90400 Belfort, France; 9 Department of Orthopedic Surgery, Humanitas Research Hospital, 20089 Milan, Italy; 10 Department of Physical Medicine and Rehabilitation, Istanbul Faculty of Medicine, Istanbul University, 34093 Istanbul, Turkey; 11 Joint and Bone Research Unit, IIS-Fundacion Jimenez Diaz UAM, 28040 Madrid, Spain; 12 Department of Medical and Surgical Specialties and Dentistry, University of Campania ‘Luigi Vanvitelli’, 81100 Caserta, Italy; 13 Department of Internal Medicine and Rheumatology, University of Medicine & Pharmacy “Carol Davila”, 050474 Bucharest, Romania; 14 Department of Orthopedic Surgery, Johanna Etienne Hospital, 41462 Neuss, Germany; 15 Physical Rehabilitation Medicine Department, Medical Rehabilitation Centre, Rovisco Pais, 3060-586 Tocha, Portugal; 16 Faculty Medicine, University of Coimbra, 3004-531 Coimbra, Portugal; 17 EUPATI Graduate and Patient Advocate, 1350-410 Brussels, Belgium; 18 Department of Rheumatology, Belorussian State Medical University, 220116 Minsk, Belarus; 19 Department of Experimental and Clinical Medicine, University of Florence, 50121 Florence, Italy; 20 Institute of Rheumatology, 12800 Prague, Czech Republic; 21 Department of Family Practice and Division of Sport and Exercise Medicine, University of British Columbia, Vancouver, BC V6Z 3B7, Canada; 22 Department of Orthopaedics and Traumatology, Fondazione Policlinico Tor Vergata, 00133 Rome, Italy; 23 Center for Treatment Comparison and Integrative Analysis, Tufts University School of Medicine, Boston, MA 02116, USA

**Keywords:** knee osteoarthritis, intra-articular hyaluronic acid, innovative, consensus

## Abstract

Background: Intra-articular hyaluronic acid (IAHA) products are often used in the treatment of adults with mild-to-moderate knee osteoarthritis (KOA). The International Symposium on Intra-Articular Treatment (ISIAT) convened a multidisciplinary technical expert panel to define characteristics for an innovative IAHA product that should answer unmet needs in the clinical management of adults with mild-to-moderate KOA. Methods: An initial set of evidence-based statements was developed based on data extracted from articles identified through a comprehensive literature search. A Delphi panel comprising 19 experts in KOA voted in 3 rounds to rate their degree of agreement with accepted statements. Results: The final set of 13 accepted statements focus on the effect of an innovative IAHA across 5 key domains of nociceptive pain, joint function, quality of life, joint structure and integrity, and adverse effects. The statements set thresholds for clinically meaningful improvements that exceed those generally achievable by currently available IAHA products. Conclusion: The characteristics described by these statements from the ISIAT set new standards for what should be expected from an innovative IAHA. These statements should serve as a framework for driving the development of innovative IAHA products that will surpass the actual outcomes achieved by current viscosupplements in patients with mild-to-moderate KOA.

## 1. Introduction

Knee osteoarthritis (KOA) has a global prevalence of nearly 24% in the adult population [1] and is a major contributor to disability [2]. The management of patients with mild-to-moderate KOA focuses initially on nonpharmacological and pharmacological strategies that can improve pain, stiffness, and function of the affected joints as well as the overall quality of life (QoL), while surgical approaches are generally reserved for patients with recurrent or persistent symptoms and disease progression [3,4,5,6,7,8,9].

Viscosupplementation stands as a well-established conservative treatment approach for KOA. A diverse range of products have been employed for this treatment modality, including corticosteroids, hyaluronic acid, growth factor/platelet-rich plasma (PRP), and mesenchymal stem cells (MSCs) [10,11]. In recent years, intra-articular injections of carboxymethyl chitosan [12] and polyacrylamide hydrogel [13] have emerged as novel and innovative therapeutic options. Despite the introduction of these new products for joint viscosupplementation, intra-articular hyaluronic acid (IAHA) remains a mainstay due to a wealth of studies involving large cohorts and extended follow-up periods. IAHA is particularly suitable for patients who fail to achieve adequate pain relief from oral medications (NSAIDs, acetaminophen), exercise, or physical therapy, as well as those with renal or gastrointestinal intolerance to NSAIDs. Apart from acute inflammation within the joint space, there are no absolute contraindications for IAHA injections. However, the drug’s effectiveness may be diminished in the presence of certain conditions, such as extensive bone edema, and acute conditions such as gout and scleroderma [1].

At least 30 different IAHA products have been used in the past few decades as nonsurgical approaches for the treatment of adults with mild-to-moderate KOA [14]. In most series, patients receiving IAHA presented with visual analog scale (VAS) ≥ 40 mm, Lequesne index ≥ 10, and Kellgren–Lawrence (KL) grade II–III [15,16,17]. In general, guidelines recognize that IAHA provides beneficial effects on pain for at least a few months, with a very good safety profile.

Recommendations regarding IAHA use are diverse and sometimes controversial, refs. [1,5,9,16,18,19,20] with some current guidelines even advising against hyaluronic acid products for viscosupplementation because of equivocal evidence found in the literature [3,21]. There is little consensus across studies regarding endpoints for measuring clinical benefit [22], and there is no consensus on the characteristics of an IAHA product that might best achieve such endpoints [23].

The International Symposium on Intra-Articular Treatment (ISIAT) convened a multidisciplinary technical expert panel to define a set of characteristics for an innovative IAHA for adults with mild-to-moderate KOA, along with threshold targets for clinically meaningful improvements in pain, joint function, and QoL that might be achieved by such products. We present a set of 13 statements accepted by the expert panel that can be used to develop an innovative IAHA product for KOA (Table 1). This article discusses the characteristics that should be present in a product to address the unmet needs in current clinical practice and how such products might best be used in the management of adults with mild-to-moderate KOA. The statements proposed focus on characteristics that drive clinical decision-making rather than the technical characteristics of IAHA that are considered in research settings.

## 2. Methods

Between September and November 2022, a MEDLINE/PubMed search was conducted for full-text guidelines, meta-analyses, and systematic literature reviews published in English since 2000 on the use of IAHA in adults with mild-to-moderate KOA using MeSH terms and free-text searches for “osteoarthritis”, “knee”, “hyaluronic acid”, “viscosupplementation”, and related words. As patients with KL IV and other indicators of severe KOA typically show minimal improvement with IAHA [24], articles specifically focused on this population were not included. Articles with duplicate information were discarded, while others were added based on a review of the references of included articles. Further searches were conducted to identify additional open-label studies and randomized controlled trials (RCTs) on specific topics not fully addressed in the main set. Data were extracted from the final set of 77 articles, with a special focus on studies identified as having crossed minimally clinically important difference thresholds [25,26]. An initial set of 27 evidence-based statements was developed based on the collected data and presented to the Steering Committee for review.

In January 2023, the Steering Committee convened a Delphi panel comprising 21 experts in KOA from 12 countries representing 6 specialties: 11 rheumatologists, 5 orthopedic surgeons, 1 epidemiologist, 2 rehabilitation medicine physicians, 1 internal medicine physician, and 1 patient representative. In the first round of online voting, panel members were asked to agree or disagree with each statement. Statements supported by ≥75% positive votes were accepted and statements with ≤67% positive answers were rejected. Statements with a positive vote between 67% and 75% were discussed and revised to address panel member concerns. In the second round of voting, panel members were asked to agree or disagree with each revised statement; those supported by ≥50% positive votes were accepted and those with ≤50% positive responses were rejected. In a third round of voting, panel members were presented with the final set of accepted statements and were asked to assign a score rating their degree of agreement on a scale from 1 to 10, where 1 represented complete disagreement and 10 represented complete agreement. Data from the three rounds of voting were then collected along with comments from panel members that arose during the discussions.

## 3. Results

The initial set of 27 statements presented to the Steering Committee described the features of an innovative IAHA product in eight domains: improvement in pain, function, QoL, and Outcome Measures in Rheumatology (OMERACT)/Osteoarthritis Research Society International (OARSI) response; delay in time to total knee arthroplasty (TKA); improvement in joint structure and integrity; and adverse effects.

In discussions among Steering Committee members, overlapping statements were collapsed and a set of 14 statements was developed for presentation to the ISIAT expert panel. After two rounds of voting, thirteen statements across five domains were accepted, including one statement that was rejected in the first round of voting but accepted in revised form in the second round of voting. In the third round of voting, the mean agreement rating for all statements was 8.8 (range, 8.2 to 9.4). This indicates an overall strong agreement with the final set of 13 statements, although some panelists rated moderate or minimal agreement with a few statements.

### 3.1. Reduction in Nociceptive Pain

The panel accepted four statements regarding the effect of an innovative IAHA on nociceptive pain in patients with mild-to-moderate KOA. The first two statements address the degree of pain reduction and the time of benefit onset after IAHA injection; the third and fourth statements address the duration of benefit and, consequently, the duration of time between injections for continued benefit.

1. An innovative IAHA should improve nociceptive pain by 50–70% as measured on a numeric rating scale or Western Ontario and McMaster Universities Arthritis Index (WOMAC) pain subscales in at least 70% of patients with mild-to-moderate KOA.

In studies of adults aged 40–80 years with mild-to-moderate KOA, 32–64% show an overall improvement in pain from baseline [4,7,24,27,28] and approximately half of the patients achieve 50% improvement [15,20,29,30,31,32]. As the prevalence of KOA is considerably lower in younger patients [2], effect of IAHA in patients under age 40 is less commonly studied. Nevertheless, available data suggest these patients show the same magnitude of benefit [15,27,33,34]. The statement accepted by the panel therefore defines a threshold for pain improvement at the upper end of what is seen with currently available agents on patient-reported numeric rating scales and WOMAC index subscales, without distinguishing between older and younger patients. The mean agreement rating for this statement was 8.9, with 16/18 panelists (89%) rating strong agreement (of ≥8), while 2/18 (11%) rated moderate agreement (of 5–7).

2. An innovative IAHA should produce initial benefit on nociceptive pain within 1 to 2 weeks in at least 70% of patients with mild-to-moderate KOA.

Studies that include an early evaluation timepoint demonstrate improvement at 4–6 weeks after injection [15,31,32,35]. The statement accepted by the panel thus represents a considerably shortened time to benefit onset. The mean agreement rating for this statement was lower than for statement 1, at 8.4; 13/18 panelists (72%) rated strong agreement (≥8), 2/18 (11%) rated moderate agreement (5–7), and 3/18 (17%) rated minimal agreement (of ≤4).

3. An innovative IAHA should maintain a beneficial effect on nociceptive pain for 9 to 12 months.

Improvement in pain after IAHA injection increases over time before slowly wearing off. Specifically, maximum improvement is generally achieved at 2–3 months and is sustained for at least 6 months, with a smaller effect seen for 12 months in some patients [3,5,20,27,29,30,33,34,35,36,37]. Thus, the statement accepted by the panel supports a longer benefit for IAHA than is generally seen in most patients. The mean agreement rating was 8.9, with 15/18 (83%) rating strong agreement (≥8) and 3/18 (17%) rating moderate agreement (6 or 7).

4. Injection of an innovative IAHA should be repeated after 9 to 12 months if the patient again becomes symptomatic.

The statement accepted by the panel fits with the standard practice of repeating the injections only if the patient is symptomatic. The mean agreement rating was 8.9, with 16/18 (89%) rating strong agreement (≥8), 1 panelist rating moderate agreement (7), and 1 panelist rating minimal agreement (4).

### 3.2. Function

The panel accepted four statements regarding the effect of an innovative IAHA on joint function in patients with mild-to-moderate KOA. Although far more studies report pain outcomes than function or stiffness [38], the same types of evaluations are typically reported. Thus, the statements regarding the effect of IAHA on function mirror those addressed for pain: The first two statements address the degree of joint function improvement and the time of improvement onset after IAHA injection, while the third and fourth statements address the duration of improvement and duration of time between injections for continued benefit.

5. An innovative IAHA should improve function by 50–70% as measured on the WOMAC subscale and the Lequesne index in patients with mild-to-moderate KOA.

RCTs measuring the effect of IAHA on WOMAC stiffness or physical activity subscales and the Lequesne index show an improvement of 40–54% from baseline in adults with mild-to-moderate KOA [30,34].

The panel accepts the statement, thereby establishing a benchmark for functional improvement, set at the same value specified in “statement 1”, beyond what is observed with currently existing agents. Nevertheless, the mean agreement rating for this statement was high at 9.1, with 16/18 panelists (89%) rating strong agreement (≥8) and 2/18 (11%) rating moderate agreement (7).

6. An innovative IAHA should produce initial benefit on joint function within 1 to 2 weeks in at least 70% of patients.

Few studies report the early effect of IAHA on joint function, but available data show some improvement by 5–6 weeks after injection [39,40]. Thus, the statement accepted by the panel represents a considerably shortened time to benefit onset. The mean agreement rating for this statement was lower at 8.2; 14/18 panelists (78%) rated strong agreement (≥8) while 4/18 (22%) rated minimal agreement (≤4).

7. An innovative IAHA should maintain a beneficial effect on joint function for 9 to 12 months.

Following IAHA injection with currently available products, improvement in function/stiffness can be maintained for up to 9–12 months. However, a return toward baseline is seen in some patients after 6 months [29,30,34,37,41,42]. The statement accepted by the panel thus supports a longer benefit for IAHA than might be seen in most patients. The mean agreement rating was 8.8, with 16/18 (89%) rating strong agreement (≥8) and 2/18 (11%) rating minimal agreement of (4).

8. Injection of an innovative IAHA should be repeated after 9 to 12 months if the patient again becomes symptomatic and requires functional improvement.

The statement accepted by the panel fits with the standard practice of repeating the injections only if the patient is symptomatic, and is supported by data showing that multiple injection cycles allow for continued beneficial effects on both pain and function [43,44]. The mean agreement rating for this statement was 9.1, with 17/18 (94%) rating agreement (≥8), and 1 panelist rating minimal agreement (4).

### 3.3. Quality of Life

The three statements accepted by the panel related to the effect of an innovative IAHA on patient QoL were similar to those related to both pain and function: The first statement addressed the degree of QoL improvement, the second addressed the duration of improvement, and the third addressed the optimal duration of time between injections to maintain benefit. Unlike statements related to pain and function benefits with IAHA, the ISIAT expert panel did not consider a separate statement on the time to onset of QoL benefit after injection.

9. An innovative IAHA should improve the perception of quality of life by 30–50% in patients with mild-to-moderate KOA.

Measures of QoL show that most adults of all ages with mild-to-moderate KOA can achieve at least 20% improvement after IAHA injection using tools such as OARSI global assessment, SF-36, and EuroQoL [15,29,36,44]. Thus, the statement accepted by the panel defines a QoL benefit threshold for an innovative IAHA above what is seen with currently available agents. The mean agreement rating for this statement was somewhat lower at 8.7; 14/18 panelists (78%) rated strong agreement (≥8), 3/18 (17%) rated moderate agreement (6–7), and 1 panelist rated minimal agreement (3).

10. An innovative IAHA should maintain a beneficial effect on the perception of quality of life for 9 to 12 months.

Studies with longer-term follow-up show that improvement in QoL after IAHA injection can be seen at 3 months and maintained for up to 9–12 months [15,36,45]. The statement accepted by the panel is thus largely in line with the QoL benefit seen with currently available IAHA products. Nevertheless, the mean agreement rating for this statement was 8.7, with 15/18 (83%) rating strong agreement (≥8), 1 panelist rating moderate agreement (7), and 2 panelists rating minimal agreement (4).

11. Injection of an innovative IAHA should be repeated after 9 to 12 months if required to maintain an appropriate quality of life.

As with the effect of IAHA injection on pain and function, additional injection cycles allow for continued beneficial effects on QoL [44]. The statement accepted by the panel is in line with the standard practice of repeating the injection if the patient is symptomatic, although this is usually defined by overall pain and function, not specifically by QoL. The mean agreement rating with this statement was 9.1, with 16/18 (89%) rating strong agreement (≥8), 1 panelist rating moderate agreement (7), and 1 panelist rating minimal agreement (4).

### 3.4. Joint Structure and Integrity

Studies exploring mechanisms driving IAHA benefit have focused on IAHA-induced changes in cartilage that might promote a slowing in structural disease progression [46,47,48,49], which, in theory, could contribute to a delay in time to TKA [50,51,52,53]. The panel considered two statements related to the effect of an innovative IAHA on joint structure and integrity. A statement that IAHA should delay time to TKA was rejected in the first round of voting. A statement that IAHA should be beneficial the natural history of the disease, including joint structure, required reformulation before passing the threshold for acceptance in the second round.

12. An innovative IAHA should demonstrate beneficial effect on slowing the progression of the disease.

Increased urine and serum biomarkers of collagen degradation indicative of increased cell turnover have been demonstrated after IAHA injection, potentially indicating improved joint structure and integrity [46,47,48,49]. IAHA promotion of anti-inflammatory responses and suppression of pro-inflammatory cytokines may further contribute to improved joint integrity [54]. Some clinical trials have shown a potentially beneficial effect on joint structure after IAHA injections [55,56]. These data broadly support the statement accepted by the ISIAT expert panel. However, links between these mechanisms and disease progression are somewhat weak. Thus, although the mean agreement rating with this statement was 8.8, with 15/18 (83%) rating strong agreement (≥8) and 2/18 (11%) rating moderate agreement (6 or 7), 1 panelist rated strong disagreement (of 1).

### 3.5. Adverse Effects

13. An innovative IAHA should be associated with local reactions in fewer than 5% of patients and with no systemic adverse effects, even after multiple injections.

Meta-analyses show an overall incidence of 8.5% for local reactions in patients treated with IAHA, ranging from a low of 6% for local skin reactions to a high of 12% for injection-site pain [57]. The statement accepted by the expert panel sets the upper boundary of tolerable adverse effects at the lowest rates reported, and does not consider a need to balance the number of injections required for treatment efficacy with improved tolerability profile, even though it can be challenging to differentiate treatment-related adverse effects from those related to the injection itself. The mean agreement rating was the highest among all statements at 9.4, with 17/18 (94%) rating strong agreement (≥8); of these, 14/18 (78%) rated complete agreement (10) and 2/18 (11%) rated strong agreement (8). The remaining panelist rated minimal agreement (4).

## 4. Discussion

The 13 statements presented here describe the characteristics of an innovative IAHA that would meet threshold targets yielding clinically meaningful benefits in adults with mild-to-moderate KOA. All these targets for improvement in pain, function, QoL, and joint integrity and for minimizing adverse effects exceed those generally achievable by currently available IAHA products, and therefore set new standards for what we expect from an innovative IAHA.

The statements focus on characteristics that drive clinical decision-making rather than technical characteristics of IAHA that are considered in research settings. For example, current evidence seems to show that high molecular weight (MW) IAHA products yield greater improvements in pain scores as well as joint function compared with low MW and/or medium MW IAHA [3,4,7,25,58,59,60,61], and products from a bacterial rather than an avian source have been associated with lower rates of local injection site reactions [58]. As the value of such distinctions is their effect on efficacy and tolerability, we chose to consider mainly their clinical impact.

The statements are presented within separate domains of pain, joint function, QoL, joint integrity, and adverse effects. However, an innovative IAHA that achieves a durable effect on pain and function is likely to be quantified as an improvement in QoL, which, in turn, will impact a decision about repeat injections and consideration for delay of TKA [52,53,62]. Thus, although the highest rating of agreement was recorded for the statement advocating adverse effects in only 5% of patients regardless of the number of injections, it is possible that higher rates up to 10–15% might be acceptable if improvements in pain, function, and QoL allow patients to benefit from repeated injections and delay time to TKA. Considering the interconnectedness of these different factors, patient advocacy groups could be particularly helpful in determining how to assess outcomes, and a tool such as the patient acceptable symptom state (PASS) [63] might prove particularly valuable in assessing the efficacy of an innovative IAHA.

Interestingly, the statement regarding IAHA delaying time to TKA was the one statement rejected by the panel. As a measure of improved joint integrity, time to TKA would be difficult to assess prospectively. There remains a lack of standardization in biochemical markers and imaging findings to demonstrate the progression of the disease, and the study would need to follow patients over many years and weigh up a multitude of potentially confounding factors such as age and functional demand. Alternatively, delay in TKA could be viewed as a measure of improved patient outcomes overall from IAHA injection [64]. In this sense, the potential for delay in TKA could be part of clinical decision-making for patients responding well to an innovative IAHA that achieves the proposed target thresholds.

Panelists showed the lowest rates of agreement with statements that support demonstrating clinical benefit at 1–2 weeks after IAHA injection. Guidelines currently recommend intra-articular corticosteroid for short-term pain relief [3,21], and trials of IAHA combined with dexamethasone, triamcinolone, or betamethasone show improvement in pain, joint function, and QoL at 1–3 weeks after injection, with no additional adverse effects and no diminishment of longer-term IAHA benefit [45,65,66]. Although the combination of steroids and IAHA might be sufficient for most patients, an innovative IAHA that meets an early benefit threshold could be particularly useful in patients undergoing repeat injections who require minimal exposure to steroids because of comorbid conditions.

We recognize the limitations of our approach in developing these statements and specifically in defining the patient population likely to be treated with the innovative IAHA described. First, we are assuming the patient is being treated for the first time with IAHA. Patients being considered for IAHA injection because of recurrent or persistent symptoms may be allowed a lower threshold for clinical benefit or may be allowed a higher threshold for tolerating adverse effects. Similarly, we are assuming the patient is being treated for unilateral KOA and receives an IAHA injection in the affected joint. Patients with bilateral KOA may find that local pain and/or functional improvement in one joint impacts QoL, but a different threshold for overall QoL benefit would be required. Importantly, in all cases, we are assuming that any benefit is due to the treatment and not to a placebo effect from the injection. Patient-reported levels of pain, stiffness, and function are all significantly improved with placebo injections, and could confound estimated benefits from the treatment itself [67]. As a wide variety of factors might influence benefit, including such a placebo effect, the physician’s ability to perform the procedure correctly, and the environment in which the procedure is performed [68], we have focused only on the beneficial effect of the treatment itself.

In discussing the accepted statements, panelists raised several important issues that remain to be addressed. Can we define characteristics of patients more likely to respond well to an innovative IAHA? Can we define a minimum number of target thresholds that an innovative IAHA should be able to achieve? Should cost and cost-effectiveness be additional factors for consideration? Should clinically relevant technical characteristics such as ease of use be considered? Most importantly, could the use of an innovative HA improve patient compliance with nonpharmacologic management recommendations such as exercise, increasing activity level, or weight loss by reducing pain and functional impairment? This last point is critical, as an innovative IAHA should not supplant nonpharmacological recommendations, but should instead be part of an overall patient management strategy. Addressing these questions and exploring other important issues must be a key part of our research agenda moving forward.

## 5. Conclusions

The 13 statements accepted by the ISIAT expert panel describe characteristics of an innovative IAHA product that would meet or exceed current clinical benefit thresholds for patients with mild-to-moderate KOA. Such an innovative IAHA would yield significant improvements in pain, joint function, QoL, and joint integrity in most patients, providing both a rapid onset of benefit and sustained benefit for a prolonged duration while minimizing adverse effects. Our goal is for these statements to serve as a backbone for driving the development of innovative IAHA products that will maximize outcomes in patients with mild-to-moderate KOA.

## Figures and Tables

**Table 1:** Characteristics of an innovative IAHA product for mild-to-moderate KOA.

Nociceptive Pain	
1	An innovative IAHA should improve nociceptive pain by 50–70% as measured on a numeric rating scale or WOMAC pain subscales in at least 70% of patients with mild-to-moderate KOA.
2	An innovative IAHA should produce initial benefit on nociceptive pain within 1 to 2 weeks in at least 70% of patients with mild-to-moderate KOA.
3	An innovative IAHA should maintain a beneficial effect on nociceptive pain for 9 to 12 months.
4	Injection of an innovative IAHA should be repeated after 9 to 12 months if the patient again becomes symptomatic.
Function	
5	An innovative IAHA should improve function by 50–70% as measured on the WOMAC subscale and the Lequesne index in patients with mild-to-moderate KOA.
6	An innovative IAHA should produce initial benefit on joint function within 1 to 2 weeks in at least 70% of patients.
7	An innovative IAHA should maintain a beneficial effect on joint function for 9 to 12 months.
8	Injection of an innovative IAHA should be repeated after 9 to 12 months if the patient again becomes symptomatic and requires functional improvement.
Quality of Life	
9	An innovative IAHA should improve the perception of quality of life by 30–50% in patients with mild-to-moderate KOA.
10	An innovative IAHA should maintain a beneficial effect on the perception of quality of life for 9 to 12 months.
11	Injection of an innovative IAHA should be repeated after 9 to 12 months if required to maintain an appropriate quality of life.
Joint Structure and Integrity	
12	An innovative IAHA should demonstrate beneficial effect on slowing the progression of the disease.
Adverse Effects	
13	An innovative IAHA should be associated with local reactions in fewer than 5% of patients and with no systemic adverse effects, even after multiple injections.

IAHA, intra-articular hyaluronic acid; KOA, knee osteoarthritis; WOMAC, Western Ontario and McMaster Universities Arthritis Index.

## Data Availability

No new data were created or analyzed in this study. Data sharing is not applicable to this article.
